# Systemic Immune-Inflammation Index Is Superior to Neutrophil to Lymphocyte Ratio in Prognostic Assessment of Breast Cancer Patients Undergoing Neoadjuvant Chemotherapy

**DOI:** 10.1155/2020/7961568

**Published:** 2020-12-18

**Authors:** Cong Jiang, Yubo Lu, Shiyuan Zhang, Yuanxi Huang

**Affiliations:** Department of Breast Surgery, Tumor Hospital Affiliated to Harbin Medical University, Harbin 150086, China

## Abstract

**Results:**

SII, NLR, and PLR did not define patient groups with distinct clinicopathological characteristics. SII, NLR, and PLR cut-off values were 547, 2.13, and 88.23, as determined by ROC analysis; the corresponding areas under the curve (AUCs) were 0.625, 0.555, and 0.571, respectively. Cox regression models identified SII as independently associated with OS. Patients with low SII had prolonged OS (65 vs. 41 months, *P* = 0.017, HR: 3.24, 95% CI: 1.23-8.55). In the *Z* test, the difference in AUC between SII and NLR was statistically significant (*Z* = 2.721, 95% CI: 0.0194-0.119, *P* = 0.0065).

**Conclusion:**

Our study suggests that the pretreatment SII value is significantly correlated with OS in breast cancer patients undergoing NAC and that the prognostic utility of SII is superior to that of NLR and PLR.

## 1. Introduction

Breast cancer is the most frequently diagnosed cancer in women and is the leading cause of female cancer death [1]. Although hereditary breast cancer accounts for 5% to 10% of breast cancer cases, nongenetic factors represent the principal factor for the differences in breast cancer incidence among different ethnic groups [2]. Risk factors for breast cancer include menstruation (early menarche age, late age of menopause), childbearing history (childless, late childbearing age, and fertility), use of exogenous hormones (oral contraceptive or hormone replacement therapy), and nutrition and anthropometry (weight, body fat centripetal distribution). Moreover, breastfeeding and physical activity are widely recognized as protective factors against breast cancer [3]. Survival rates for patients have improved dramatically due to advances in treatment strategies [4]. However, 20%-25% of patients are diagnosed with locally advanced breast cancer, which is associated with a higher rate of recurrence and patient mortality [5, 6].

Neoadjuvant chemotherapy (NAC), as the standard treatment for locally advanced and inoperable tumors, has been widely used in breast cancer patients. The main aim of NAC use is to improve tumor operability, reduce tumor size, and improve the likelihood of eligibility for breast-conserving management strategies [7, 8]. Molecular subtypes and biomarkers such as Ki-67 and residual cancer burden have been used to predict overall survival (OS) following NAC; however, no further markers are used to routinely aid prognostication.

In recent years, the immune system has been recognized to play an essential role in breast cancer treatment response [9, 10]. As key components of the host immune system, inflammatory biomarkers such as neutrophil (N), lymphocyte (L), and platelet (P) levels—alongside neutrophil-to-lymphocyte ratio (NLR) and platelet-to-lymphocyte ratio (PLR)—have been identified as prognostic factors for many other malignant tumors [11–15].

As a comprehensive hematological parameter, the systemic immune-inflammation index (SII), which is based on neutrophil (N), platelet (P), and lymphocyte (L) counts (SII = N × P/L), can better reflect the immune and inflammatory state of the body compared to the use of any one of these markers in isolation [16]. Although SII has been used to study different cancers, including liver, pancreatic, and gastric cancers [16–18], it has not been widely investigated in the context of breast cancer. This article is aimed at exploring the prognostic role of SII in breast cancer patients undergoing neoadjuvant chemotherapy.

## 2. Materials and Methods

### 2.1. Patient Selection

Between January 2014 and May 2018, a total of 249 breast cancer patients received NAC and underwent surgery at the Tumor Hospital Affiliated to Harbin Medical University, Harbin, China. The study was approved by the ethics committee of the Tumor Hospital Affiliated to Harbin Medical University, including confirmation of adherence to the Declaration of Helsinki and its later amendments. All patients gave written informed consent before study participation.

The eligibility criteria for the patients included (i) pathological confirmation of breast cancer diagnosis by core needle biopsy prior to NAC treatment, (ii) consent for NAC and surgery in our hospital, (iii) complete clinical recorded information and follow-up data, and (iv) peripheral blood samples obtained within one week prior to NAC initiation.

The exclusion criteria for the patients included (i) receipt of radiotherapy, endocrine therapy, or targeted therapy prior to NAC; (ii) diagnosis of chronic inflammatory or autoimmune disease, such as liver cirrhosis or systemic lupus erythematosus (SLE); and (iii) patients with distant metastasis.

### 2.2. Chemotherapy Regimens

All patients received NAC after diagnosis; the chemotherapy regimen was selected according to the results of immunohistochemistry and patients' wishes. Anthracycline- (A-) based and/or taxane- (T-) based NAC regimens were used in our study; AC regimen: anthracyclines 90 mg/m^2^ and cyclophosphamide (C) 600 mg/m^2^; AC-T regimen: anthracyclines 90 mg/m^2^, cyclophosphamide 600 mg/m^2^, and taxanes 80-100 mg/m^2^; AC-TH regimen: anthracyclines 90 mg/m^2^, cyclophosphamide 600 mg/m^2^, taxanes 75 mg/m^2^, and Herceptin (H) first dose 8 mg/kg, then 6 mg/kg; and TAC regimen: taxanes 75 mg/m^2^, anthracyclines 50 mg/m^2^, and cyclophosphamide 500 mg/m^2^. One cycle of the chosen regimen was repeated every 3 weeks. All patients received at least four cycles of NAC. Patients with docetaxel were given dexamethasone and diphenhydramine before chemotherapy to prevent allergy. Surgery was performed following approximately two weeks of rest period after NAC completion, according to the patient's condition.

### 2.3. Classification Criteria

The TNM stage system was performed according to the eighth edition of the American Joint Committee on Cancer (AJCC) [19]. Luminal A, luminal B/HER-2-positive, luminal B/HER-2-negative, triple negative, and HER-2-enriched molecular subtypes were used [20]. Age and N, L, and P count groups were divided by the median. High and low SII, NLR, and PLR groups were identified using thresholds determined by receiver operating characteristic (ROC) analysis of maximum sensitivity and specificity.

### 2.4. Follow-Up

All patients received a 3-monthly follow-up for two years after surgery, a 6-monthly follow-up for the next three years, and then an annual follow-up. Laboratory tests, physical examination (breast and lymph node palpation), breast ultrasonography, liver ultrasound, mammography, and other suitable examinations are used to assess the physical condition of patients at follow-up.

### 2.5. Statistical Analysis

Statistical analyses were conducted by SPSS software (version 17.0) and MedCalc software (version 19.0.7). ROC analysis was used to determine the optimal cut-off value for patient dichotomization thresholds. Categorical variables are described using frequencies and percentages (%); differences were assessed by a chi-squared test or Fisher's exact test. OS time was determined using the Kaplan-Meier product limit estimator method; survival associations were determined by Kaplan-Meier plots and log-rank test. Cox proportional hazards regression models were used to examine the independence of prognostic factors. *Z* tests were used to compare the difference in predictive ability between different groups. *P* < 0.05 was considered statistically significant.

## 3. Results

### 3.1. Clinicopathologic Characteristics of all Breast Cancer Patients Divided by SII, NLR, and PLR

Two hundred and forty-nine breast cancer patients were classified by SII, NLR, and PLR. All cases were female; the median patient age was 51 years. BMI was calculated and classified according to WHO criteria: under normal weight (BMI < 18.5; 8 patients, 3.2%), normal weight (BMI ≥ 18.5; 154 patients, 61.8%), overweight (BMI ≥ 25; 71 patients, 28.5%), and obese (BMI ≥ 30; 16 patients, 6.4%). 50 patients (20.0%) achieved complete pathological response (pCR).

The clinicopathological characteristics of patients in different SII, NLR, and PLR groups are shown in Tables 1–3. ROC analysis was used to determine the optimal cut-off value for patient grouping by SII (547 × 10^9^/L), NLR (2.13 × 10^9^/L), and PLR (88.23 × 10^9^/L). Hence, patients were dichotomized by these markers: low SII (<547 × 10^9^/L, 183 patients, 73.5%) and high SII (≥547 × 10^9^/L; 66 patients, 26.5%), low NLR (<2.13 × 10^9^/L; 177 patients, 71.1%) and high NLR (>2.13 × 10^9^/L; 72 patients, 28%), and low PLR (<88.23 × 10^9^/L; 49 patients, 19.7%) and high PLR (>88.23 × 10^9^/L; 200 patients, 80.3%).

Patient groups defined by SII, NLR, and PLR demonstrated similar clinicopathological characteristics (Tables 1–3). Low SII value was significantly associated with the HER-2 subgroup (*X*^2^ = 4.019, *P* = 0.045) and low NLR (*X*^2^ = 4.879, *P* = 0.027). PLR status was significantly associated with patient age (*X*^2^ = 5.552, *P* = 0.018).

### 3.2. Cox Regression Survival Analyses

**Figure 1:**
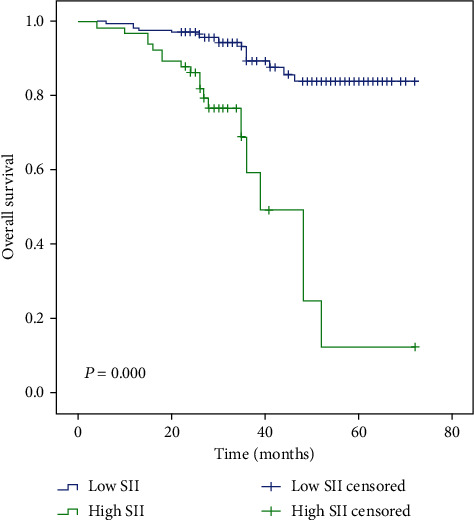
Kaplan-Meier analysis of OS in patients of high and low SII with breast cancer.

**Figure 2:**
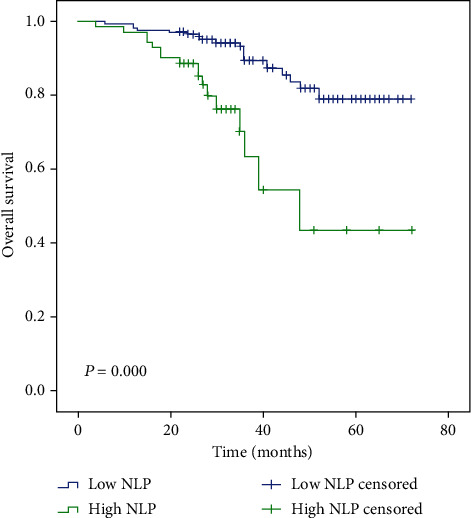
Kaplan-Meier analysis of OS in patients of high and low NLR with breast cancer.

**Figure 3:**
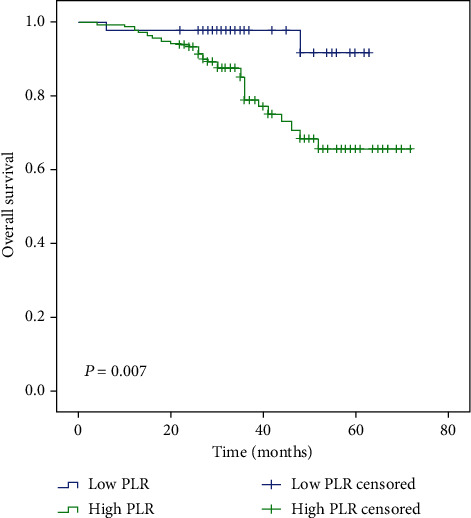
Kaplan-Meier analysis of OS in patients of high and low PLR with breast cancer.

Follow-up time ranged from 4 to 72 months; 5 patients were followed up less than 12 months. The median follow-up time was 34 and 28 months in the low and high SII groups, respectively. The median follow-up time of low NLR, high NLR, low PLR, and high PLR groups was 34, 28, 35, and 32 months, respectively. Median OS time was only reached in the high SII and high NLR groups (39 months and 48 months, respectively). However, the mean OS time in patients with low SII, low NLR, and low PLR is significantly longer than that in those patients with high SII, high NLR, and high PLR, as determined by the log-rank test (65 vs. 41 months, *P* ≤ 0.001; 64 vs. 50 months, *P* ≤ 0.001; and 61 vs. 59 months, *P* = 0.007, respectively) (Figures 1–3).

In univariate analysis, clinical T stage, ER status, PR status, molecular subtype, P, SII, NLR, and PLR were significantly associated with differential OS. However, multivariable analysis identified only SII as independently associated with OS, with the low SII group demonstrating prolonged OS time (*P* = 0.017, HR:3.24, 95% CI: 1.23-8.55) (Table 4).

### 3.3. Comparison of Prognostic Ability of SII, NLR, and PLR

**Figure 4:**
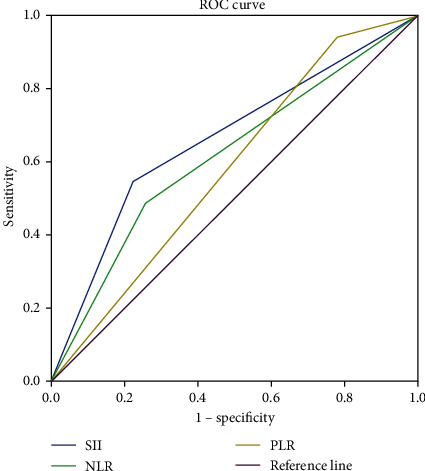
Comparison of prognostic ability of SII, NLR, and PLR.

In order to determine the value of different hematological parameters, AUC values were compared by ROC analysis. SII had a significantly higher AUC value compared to NLR and PLR (AUC = 0.625, *P* = 0.018) (Table 5 and Figure 4). In the *Z* test, the difference of AUC between SII and NLR is also statistically significant (*Z* = 2.721, 95% CI: 0.0194-0.119, *P* = 0.0065). By contrast, comparisons of SII versus PLR and NLR vs. PLR did reveal statistically significant differences (*Z* = 1.039, 95% CI: -0.0478-0.156, *P* = 0.2986; *Z* = 0.255, 95% CI: -0.103-0.134, *P* = 0.7989, respectively) (Table 6).

## 4. Discussion

We investigated the clinical significance of SII in breast cancer patients receiving NAC and compared the prognostic capacity of SII, PLR, and NLR. We demonstrate that high SII is significantly associated with shorter OS and that SII outperforms NLR as a prognostic marker in breast cancer patients receiving NAC.

Peripheral venous blood markers are known to reflect the condition of the whole immune system. The link between systemic inflammation and malignant tumors has been demonstrated in numerous studies [21–25]. Moreover, levels of neutrophils, platelets, and lymphocytes are prognostic in multiple cancer types. Neutrophils play a role in the proliferation and metastasis of tumors by releasing inflammatory mediators such as neutrophil elastase, interleukin-8, matrix metalloproteinase-9, and vascular endothelial growth factor [26–28]. Platelets can promote tumor angiogenesis and metastasis and protect tumor cells from antitumor immune response [29–31]. By contrast, lymphocytes represent a key component of the antitumor immune response, preventing tumor growth and metastasis, prolonging patient survival [32–34].

Few studies have investigated the impact of SII in breast cancer patients receiving NAC. In a study by Liu and colleagues, SII was identified as an independent prognostic factor in TNBC patients [35]. In two other studies investigating HER-2-positive breast cancer, SII was also related to OS time [36, 37]. By contrast, Chen and colleagues comprehensively evaluated the prognostic effects of SII on a nonsubtype-specific manner [38]; this study demonstrated differential 3-, 5-, and 10-year DFS and OS according to SII status [38]. In our study, high SII is related to the shorter mean OS time compared to low SII (65 vs. 41 months, *P* ≤ 0.001). Multivariate Cox regression analysis identified that SII is the only factor independently associated with survival (*P* = 0.017, HR: 3.244, 95% CI: 1.230-8.554).

A number of studies have investigated NLR and PLR; however, these have failed to produce consistent results. A meta-analysis demonstrated that high NLR was significantly associated with poor pathological response [39] but failed to demonstrate a significant association with DFS and OS in breast cancer patients. By contrast, a separate meta-analysis identified poor OS and high recurrence risk for breast cancer patients with high NLR and PLR [40]. Similarly, Bun et al. identified NLR as a significant and independent factor associated with OS time [41]. However, a separate study by Malek et al. showed that NLR was not associated with pCR [42]. In the current study, the low NLR and low PLR groups demonstrated better mean OS time (64 vs. 50 months, *P* ≤ 0.001; 61 vs. 59 months, *P* = 0.007, respectively). However, we did not identify a significant independent association between NLR or PLR and OS time upon multivariable analysis (*P* > 0.05).

We demonstrate that, compared to NLR and PLR, SII has statistically significantly higher AUC and specificity by ROC analysis (AUC = 0.625, 95% CI: 0.515-0.735, specificity = 0.780, *P* = 0.018). Pairwise comparison of SII, NLR, and PLR using *Z* tests identified a significantly higher AUC for SII than NLR (*P* = 0.0065). Together, these results suggest that SII is of greater prognostic utility than NLR.

As hematological parameters, SII, NLR, and PLR are easy to obtain and are easy to measure repeatedly; at least some of these measurements have been demonstrated—by ourselves and others—as prognostic indicators with potential clinical utility. We demonstrate that, compared with NLR and PLR, SII appears to be of the greatest independent value.

We acknowledge several limitations of our study; firstly, it is a single-center retrospective study with no control group. Secondly, our cohort has a relatively short follow-up; longer follow-up time is required to determine the impact of SII, PLR, and NLR on long-term clinical outcomes. Independent validation in a large breast cancer patient cohort is needed.

## 5. Conclusion

Our study comprehensively analyzes the prognostic value of SII in breast cancer patients undergoing NAC and compared the relative prognostic performance of SII, NLR, and PLR. Our findings suggest that the pretreatment SII is significantly associated with OS in breast cancer independent of other factors and that SII is superior to NLR and PLR. SII is therefore worthy of further investigation as a prognostic hematologic parameter for breast cancer patients.

## Figures and Tables

**Table 1:** Association between clinicopathological factors and different SII groups.

Parameters	*n* = 249 (%)	SII ≤ 547	SII > 547	*X* ^2^	*P* value
Cases		*n* = 183 (%)	*n* = 66 (%)		
Age (years)				2.493	0.114
≤51	134 (53.8%)	93 (50.8%)	41 (62.1%)		
>51	115 (46.2%)	90 (49.2%)	25 (37.9%)		
Tumor position				0.059	0.807
Left	139 (55.8%)	103 (56.3%)	36 (54.5%)		
Right	110 (44.2%)	80 (43.7%)	30 (45.5%)		
BMI				0.865	0.864
<18.5	8 (3.2%)	7 (3.8%)	1 (1.5%)		
≥18.5	154 (61.8%)	113 (61.7%)	41 (62.1%)		
≥25	71 (28.5%)	52 (28.4%)	19 (28.8%)		
≥30	16 (6.4%)	11 (6.0%)	5 (7.6%)		
Parturition				2.166	0.537
0	33 (13.3%)	26 (14.2%)	7 (10.6%)		
1	150 (60.2%)	110 (60.1%)	40 (60.6%)		
2	54 (21.7%)	36 (19.7%)	18 (27.3%)		
>2	12 (4.8%)	11 (6.0%)	1 (1.5%%)		
cT stage				4.379	0.201
T1	22 (8.8%)	17 (9.3%)	5 (7.6%)		
T2	187 (75.1%)	141 (77.0%)	46 (69.7%)		
T3	35 (14.1%)	23 (12.6%)	12 (18.2%)		
T4	5 (2.0%)	2 (1.1%)	3 (4.5%)		
ER				1.612	0.204
Negative	86 (34.5%)	59 (32.2%)	27 (40.9%)		
Positive	163 (65.5%)	124 (67.8%)	39 (59.1%)		
PR				0.613	0.434
Negative	118 (47.4%)	84 (45.9%)	34 (51.5%)		
Positive	131 (52.6%)	99 (54.1%)	32 (48.5%)		
HER-2				4.019	0.045
Negative	161 (64.7%)	125 (68.3%)	36 (54.5%)		
Positive	88 (35.3%)	58 (31.7%)	30 (45.5%)		
Ki-67				0.699	0.403
≤14%	73 (29.3%)	51 (27.9%)	22 (33.3%)		
>14%	176 (70.7%)	132 (72.1%)	44 (66.7%)		
Subtype				5.369	0.252
Luminal A	25 (10.0%)	20 (10.9%)	5 (7.6%)		
Luminal B/HER-2-	100 (40.2%)	77 (42.1%)	23 (34.8%)		
Luminal B/HER-2+	42 (16.9%)	30 (16.4%)	12 (18.2%)		
TNBC	36 (14.5%)	28 (15.3%)	8 (12.1%)		
HER-2 enriched	46 (18.5%)	28 (15.3%)	18 (27.3%)		
P53				0.327	0.567
Negative	117 (47.0%)	84 (45.9%)	33 (50.0%)		
Positive	132 (53.0%)	99 (54.1%)	33 (50.0%)		
pCR				1.804	0.179
No	199 (79.9%)	150 (82.0%)	49 (74.2%)		
Yes	50 (20.1%)	33 (18.0%)	17 (25.8%)		

Abbreviation: BMI: body mass index; cT stage: clinical T stage; ER: estrogen receptor; PR: progesterone receptor; HER-2: human epidermal growth factor receptor-2; TNBC: triple negative breast cancer; pCR: pathologic complete response.

**Table 2:** Association between clinicopathological factors and different NLR groups.

Parameters	*n* = 249 (%)	NLR ≤ 2.13	NLR > 2.13	*X* ^2^	*P* value
Cases		*n* = 177 (%)	*n* = 72 (%)		
Age (years)				1.422	0.233
≤51	134 (53.8%)	91 (51.4%)	43 (59.7%)		
>51	115 (46.2%)	86 (48.6%)	29 (40.3%)		
Tumor position				0.624	0.429
Left	139 (55.8%)	96 (54.2%)	43 (59.7%)		
Right	110 (44.2%)	81 (45.8%)	29 (40.3%)		
BMI				2.702	0.437
<18.5	8 (3.2%)	7 (4.0%)	1 (1.4%)		
≥18.5	154 (61.8%)	109 (61.6%)	45 (62.5%)		
≥25	71 (28.5%)	52 (29.4%)	19 (26.4%)		
≥30	16 (6.4%)	9 (5.1%)	7 (9.7%)		
Parturition				3.684	0.294
0	33 (13.3%)	25 (14.1%)	8 (11.1%)		
1	150 (60.2%)	106 (59.9%)	44 (61.1%)		
2	54 (21.7%)	35 (19.8%)	19 (26.4%)		
>2	12 (4.8%)	11 (6.2%)	1 (1.4%)		
cT stage				5.760	0.109
T1	22 (8.8%)	18 (10.2%)	4 (5.6%)		
T2	187 (75.1%)	136 (76.8%)	51 (70.8%)		
T3	35 (14.1%)	21 (11.9%)	14 (19.4%)		
T4	5 (2.0%)	2 (1.1%)	3 (4.2%)		
ER				0.848	0.357
Negative	86 (34.5%)	58 (32.8%)	28 (38.9%)		
Positive	163 (65.5%)	119 (67.2%)	44 (61.1%)		
PR				0.650	0.420
Negative	118 (47.4%)	81 (45.8%)	37 (51.4%)		
Positive	131 (52.6%)	96 (54.2%)	35 (48.6%)		
HER-2				4.879	0.027
Negative	161 (64.7%)	122 (68.9%)	39 (54.2%)		
Positive	88 (35.3%)	55 (31.1%)	33 (45.8%)		
Ki-67				0.788	0.375
≤14%	73 (29.3%)	49 (27.7%)	24 (33.3%)		
>14%	176 (70.7%)	128 (72.3%)	48 (66.7%)		
Subtype				5.261	0.262
Luminal A	25 (10.0%)	20 (11.3%)	5 (6.9%)		
Luminal B/HER-2-	100 (40.2%)	75 (42.4%)	25 (34.7%)		
Luminal B/HER-2+	42 (16.9%)	27 (15.3%)	15 (20.8%)		
TNBC	36 (14.5%)	27 (15.3%)	9 (12.5%)		
HER-2 enriched	46 (18.5%)	28 (15.8%)	18 (25.0%)		
P53				0.369	0.544
Negative	117 (47.0%)	81 (45.8%)	36 (50.0%)		
Positive	132 (53.0%)	96 (54.2%)	36 (50.0%)		
pCR				0.787	0.375
No	199 (79.9%)	144 (81.4%)	55 (76.4%)		
Yes	50 (20.1%)	33 (18.6%)	17 (23.6%)		

Abbreviation: BMI: body mass index; cT stage: clinical T stage; ER: estrogen receptor; PR: progesterone receptor; HER-2: human epidermal growth factor receptor-2; TNBC: triple negative breast cancer; pCR: pathologic complete response.

**Table 3:** Association between clinicopathological factors and different PLR groups.

Parameters	*n* = 249 (%)	PLR ≤ 88.23	PLR > 88.23	*X* ^2^	*P* value
Cases		49 (%)	200 (%)		
Age (years)				5.552	0.018
≤51	134 (53.8%)	19 (38.8%)	115 (57.5%)		
>51	115 (46.2%)	30 (61.2%)	85 (42.5%)		
Tumor position				0.279	0.597
Left	139 (55.8%)	29 (59.2%)	110 (55.0%)		
Right	110 (44.2%)	20 (40.8%)	90 (45.0%)		
BMI				7.153	0.052
<18.5	8 (3.2%)	4 (8.2%)	4 (2.0%)		
≥18.5	154 (61.8%)	24 (49.0%)	130 (65.0%)		
≥25	71 (28.5%)	17 (34.7%)	54 (27.0%)		
≥30	16 (6.4%)	4 (8.2%)	12 (6.0%)		
Parturition				2.166	0.537
0	33 (13.3%)	6 (12.2%)	27 (13.5%)		
1	150 (60.2%)	27 (55.1%)	123 (61.5%)		
2	54 (21.7%)	12 (24.5%)	42 (21.0%)		
>2	12 (4.8%)	4 (8.2%)	8 (4.0%)		
cT stage				5.607	0.111
T1	22 (8.8%)	7 (14.3%)	15 (7.5%)		
T2	187 (75.1%)	39 (79.6%)	148 (74.0%)		
T3	35 (14.1%)	3 (6.1%)	32 (16.0%)		
T4	5 (2.0%)	0 (0.0%)	5 (2.5%)		
ER				0.416	0.519
Negative	86 (34.5%)	15 (30.6%)	71 (35.5%)		
Positive	163 (65.5%)	34 (69.4%)	129 (64.5%)		
PR				0.323	0.570
Negative	118 (47.4%)	25 (51.0%)	93 (46.5%)		
Positive	131 (52.6%)	24 (49.0%)	107 (53.5%)		
HER-2				0.193	0.660
Negative	161 (64.7%)	33 (67.3%)	128 (64.0%)		
Positive	88 (35.3%)	16 (32.7%)	72 (36.0%)		
Ki-67				0.229	0.633
≤14%	73 (29.3%)	13 (26.5%)	60 (30.0%)		
>14%	176 (70.7%)	36 (73.5%)	140 (70.0%)		
Subtype				2.081	0.721
Luminal A	25 (10.0%)	7 (14.3%)	18 (9.0%)		
Luminal B/HER-2-	100 (40.2%)	18 (36.7%)	82 (41.0%)		
Luminal B/HER-2+	42 (16.9%)	9 (18.4%)	33 (16.5%)		
TNBC	36 (14.5%)	8 (16.3%)	28 (14.0%)		
HER-2 enriched	46 (18.5%)	7 (14.3%)	39 (19.5%)		
P53				0.933	0.334
Negative	117 (47.0%)	20 (40.8%)	97 (48.5%)		
Positive	132 (53.0%)	29 (59.2%)	103 (51.5%)		
pCR				2.334	0.127
No	199 (79.9%)	43 (87.8%)	156 (78.0%)		
Yes	50 (20.1%)	6 (12.2%)	44 (22.0%)		

Abbreviation: BMI: body mass index; cT stage: clinical T stage; ER: estrogen receptor; PR: progesterone receptor; HER-2: human epidermal growth factor receptor-2; TNBC: triple negative breast cancer; pCR: pathologic complete response.

**Table 4:** Univariate and multivariate Cox regression survival analyses of the SII for the prediction of the OS in patients with breast cancer.

Parameters	Univariate analysis	*P* value	Multivariate analysis	*P* value
	HR (95% CI)		HR (95% CI)	
Age (≤51/>51years old)	1.145 (0.590-2.224)	0.688		
Position (left/right)	1.692 (0.866-3.306)	0.124		
BMI (U+N/OW+OB)	0.910 (0.457-1.813)	0.789		
Parturition (0/≥1)	1.052 (0.408-2.716)	0.916		
cT stage (T1+T2/T3+T4)	2.115 (1.014-4.413)	0.046	1.214 (0.558-2.641)	0.624
ER (negative/positive)	0.330 (0.168-0.650)	0.001	0.526 (0.112-2.472)	0.416
PR (negative/positive)	0.305 (0.143-0.652)	0.002	0.428 (0.140-1.314)	0.138
HER-2 (negative/positive)	1.537 (0.786-3.007)	0.209		
Ki-67 (≤14%/>14%)	2.284 (0.886-5.891)	0.087		
Subtype (A/B-/B+/TNBC/HER-2)	1.527 (1.186-1.966)	0.001	0.902 (0.494-1.648)	0.738
P53 (negative/positive)	1.166 (0.593-2.291)	0.656		
pCR (no/yes)	0.843 (0.350-2.033)	0.704		
N (≤3.65/>3.65)	1.544 (0.795-3.039)	0.197		
P (≤252/>252)	2.298 (1.142-4.626)	0.020	1.288 (0.576-2.878)	0.538
L (≤2.19/>2.19)	0.733 (0.376-1.427)	0.361		
SII (≤547/>547)	6.302 (3.149-12.62)	0.000	3.244 (1.230-8.554)	0.017
NLR (≤2.13/>2.13)	4.032 (2.033-7.996)	0.000	1.769 (0.672-4.659)	0.248
PLR (≤88.23/>88.23)	5.621 (1.345-23.49)	0.018	3.539 (0.782-16.02)	0.101

Abbreviation: BMI: body mass index; U+N: under normal weight (BMI < 18.5) and normal weight (BMI ≥ 18.5); OW+OB: overweight (BMI ≥ 25) and obesity (BMI ≥ 30); cT stage: clinical T stage; ER: estrogen receptor; PR: progesterone receptor; HER-2: human epidermal growth factor receptor-2; A: luminal A; B-: luminal B/HER-2-; B+: luminal B/HER-2+; TNBC: triple negative breast cancer; HER-2: HER-2 enriched; pCR: pathologic complete response; N: neutrophil; P: platelet; L: lymphocyte; SII: systemic immune-inflammation index; NLR: neutrophil-to-lymphocyte ratio; PLR: platelet-to-lymphocyte ratio.

**Table 5:** Receiver operating characteristics analyses of SII, NLR, and PLR in patients with breast cancer.

Parameters	Cut-off value	AUC (95% CI)	Sensitivity	Specificity	*P* value
SII	547	0.625 (0.515-0.735)	0.543	0.780	0.018
NLR	2.13	0.555 (0.435-0.676)	0.486	0.748	0.293
PLR	88.23	0.571 (0.467-0.675)	0.943	0.220	0.179

**Table 6:** The difference between SII, NLR, and PLR.

Parameters	Difference of AUC	SE	95% CI	*Z* statistic	*P* value
SII vs. NLR	0.0694	0.0255	0.0194-0.119	2.721	0.0065
SII vs. PLR	0.0540	0.0520	-0.0478-0.156	1.039	0.2986
NLR vs. PLR	0.0154	0.0605	-0.103-0.134	0.255	0.7989

## Data Availability

The measurement and enumeration data used to support the findings of this study are restricted by the ethics committee of Tumor Hospital Affiliated to Harbin Medical University in order to protect patient privacy.
